# Combining Experiments and Simulations Using the Maximum Entropy Principle

**DOI:** 10.1371/journal.pcbi.1003406

**Published:** 2014-02-20

**Authors:** Wouter Boomsma, Jesper Ferkinghoff-Borg, Kresten Lindorff-Larsen

**Affiliations:** 1 Structural Biology and NMR Laboratory, Department of Biology, University of Copenhagen, Copenhagen, Denmark; 2 Cellular Signal Integration Group, Center for Biological Sequence Analysis, Technical University of Denmark, Lyngby, Denmark; Stanford University, United States of America

## Abstract

A key component of computational biology is to compare the results of computer modelling with experimental measurements. Despite substantial progress in the models and algorithms used in many areas of computational biology, such comparisons sometimes reveal that the computations are not in quantitative agreement with experimental data. The principle of maximum entropy is a general procedure for constructing probability distributions in the light of new data, making it a natural tool in cases when an initial model provides results that are at odds with experiments. The number of maximum entropy applications in our field has grown steadily in recent years, in areas as diverse as sequence analysis, structural modelling, and neurobiology. In this Perspectives article, we give a broad introduction to the method, in an attempt to encourage its further adoption. The general procedure is explained in the context of a simple example, after which we proceed with a real-world application in the field of molecular simulations, where the maximum entropy procedure has recently provided new insight. Given the limited accuracy of force fields, macromolecular simulations sometimes produce results that are at not in complete and quantitative accordance with experiments. A common solution to this problem is to explicitly ensure agreement between the two by perturbing the potential energy function towards the experimental data. So far, a general consensus for how such perturbations should be implemented has been lacking. Three very recent papers have explored this problem using the maximum entropy approach, providing both new theoretical and practical insights to the problem. We highlight each of these contributions in turn and conclude with a discussion on remaining challenges.

## Introduction

Picture this scenario: you have spent years developing an elaborate model for a particular scientific phenomenon. Now, new experimental data have been measured for the same phenomenon, and the data disagree with your model. How do you proceed? This is a reasonable question to pose in any scientific discipline, but perhaps particularly in that of computational biology, where models are constantly developed and refined to encompass the ever-growing databases of biological data.

Bayesian inference is commonly put forward as an answer to this question. It provides a simple recipe for how to produce a new model (posterior) by modifying an existing model (prior) after observing a new set of data. There are, however, situations where the Bayesian formalism is not easily applicable. For instance, it is traditionally assumed that all our prior knowledge about the measured quantities can be expressed in terms of probability distributions. Often, however, we only obtain information about the average value of these quantities from experiments. This information must somehow be turned into a probability distribution concerning the system under study before we can apply the Bayesian machinery, but intuitively it seems unreasonable to assume knowledge about an entire distribution when all we know is a single value. It is an underdetermined problem, in the sense that there may be an infinite number of possible prior distributions that are compatible with this piece of data. A simple, but general solution to this type of problem was provided by Jaynes in 1957, who proposed that among all the models fulfilling the constraints from the data, one should select the model containing the least amount of information [1]. This *maximum entropy* principle has proven to be extremely powerful, having applications in a wide variety of scientific disciplines.

In computational biology, maximum entropy approaches are also becoming increasingly common. Examples include the formulation of models of collective neural stimuli [2], reconstruction of protein signaling networks [3], optimization of force fields for molecular simulation [4], and modelling covariation among sites in protein sequences [5], [6]. The general applicability of the principle suggests that there is a significant potential for other relevant applications in this field. With this Perspectives article, we will highlight the approach in some detail, hopefully communicating the elegance of the procedure and encouraging further work in this direction.

**Figure 1:**
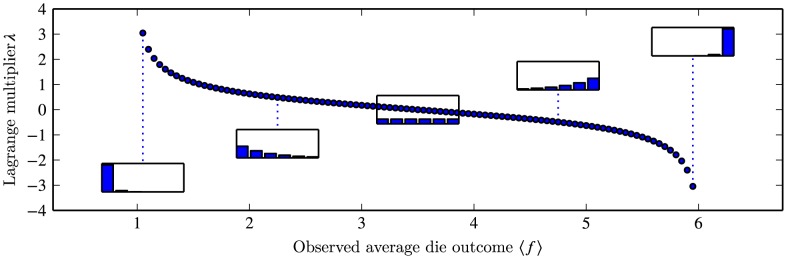
Jaynes' die problem: Maximum entropy probability distributions for a die, after observing the average outcome.

As a concrete example, we will focus our attention on a recent application in the field of structural biology, namely, the problem of conducting molecular simulations under restraints from experimental data. In this specific case, the force field can be considered as the model. Decades of research have gone into the development and fine-tuning of these force fields, and they have proven useful in a multitude of applications [7]. Figure 1 Despite their success, it is, however, still a common scenario that the results obtained through simulations do not quantitatively match those obtained from experiments. A relevant question is then how one can make use of additional information obtained through experiments to improve the quality of a simulation. Although efficient algorithms exist for improving molecular force fields based on experimental data [8], a common approach is to introduce a system-specific modification to the energy function, and thereby modify the structural ensemble to become in agreement with the experimental data. Various techniques for direct combination of experiment and simulation exist, but the theoretical underpinnings of these approaches have remained elusive. Three recent papers [9]–[11], have explored the assumptions underlying existing methods in the light of the maximum entropy principle, leading to suggestions for new avenues to optimally utilize the complementary information available from experiments and molecular simulations. We here review these developments and suggest areas that are in need of further study. In particular, we discuss the complications that may arise when using the technique in practice, including the fact that all experiments contain various, sometimes unknown, sources of noise.

## Jaynes' Principle of Maximum Entropy

Jaynes originally proposed the maximum entropy principle to establish a link between Shannon's information theory [12] and statistical mechanics [1]. The goal is to construct the probability distribution that best represents the state of knowledge after observing a set of quantities of a system. The central idea is that among all the infinite number of distributions that are compatible with the data, one should select the distribution which maintains the largest degree of uncertainty about the variables of interest, thus ensuring that the data has been used as conservatively as possible. The natural quantity for expressing uncertainty in a distribution is Shannon's entropy [12]:
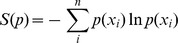
Finding the correct probability distribution thus becomes a matter of maximizing this expression under the constraint that 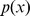 sums to one (i.e. it should be a probability distribution), combined with the constraints obtained from the observed data. Typically, the Lagrange formalism is used to enforce these constraints.

As an example, Box 1 contains a primer of the basic maximum entropy procedure on the simple problem of inferring the probability of the different outcomes of a (possibly biased) die, given only information about the average observed after a large number of throws. Following the exact same procedure, with just a few lines of calculation, the principle of maximum entropy also predicts the well-known Boltzmann distribution in statistical physics as the correct distribution reflecting your knowledge of a system when only the mean energy is observed [1]. In fact, one of Jaynes' great achievements was to demonstrate that many results in statistical mechanics could be derived by the simple application of this principle.

**Box 1. A Primer to the Principle of Maximum Entropy (Adapted from Ref. [17])**

**Jaynes' die problem**

A die has been tossed many times, and we are provided with the information that the average outcome was some value 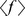, rather than the 3.5 that one would expect from a fair die. Based on this information alone, what is our estimate of the probabilities of the different outcomes for this die?

According to the Principle of Maximum Entropy, we should maximize the entropy 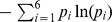 of the discrete probability distribution 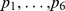. This should be done under the constraints 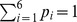 and 
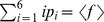
.

In general, the solution to this type of optimization problem takes the form:
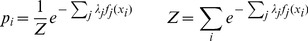
(9)where 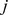 runs over the number of constraints, and Z is the partition function, which ensures proper normalization. The following identity conveniently relates the derivatives of the partition function to the observed expectation values:
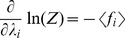
(10)For our die problem, we have only a single constraint, and since 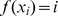, we have:
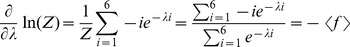
(11)from which we obtain the following five-degree polynomial in 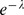:
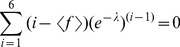
(12)
Figure 1 shows the single, real-valued solution to this polynomial for various values of 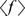. Notice how the value 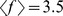 produces the uniform distribution as we expect, while higher or lower values produce gradually more skewed distributions.

In the scenario drawn up in the beginning of this article, we already have a model, and are interested in finding the necessary modifications to make it compatible with the new data. In this case, it is more convenient to consider the *relative entropy*, or *Kullback-Leibler divergence* (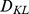) instead [13]:
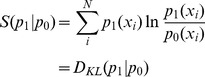
By minimizing this expression under the constraints from the experimental data, we find the distribution 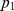 that is as close as possible to the original distribution 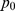, but is now compatible with the data. This procedure is sometimes referred to as the principle of *minimum discrimination information* or *minimum cross entropy*, but can be seen as a natural extension of the maximum entropy approach.

There is a substantial literature on the foundations of maximum entropy and why it is an appropriate framework for inference [1], [14]–[16]. Although a full treatment is beyond the scope of this paper, one intuitive argument for its validity comes from combinatorics: the principle of maximum entropy will provide the solution which is realizable in the most ways. For our example, if we consider all 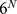 possible outcomes of 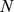 throws of a die, only a subset of these would be compatible with a given model. Maximizing the entropy ensures that this subset is as large as possible given the observed average value, not ruling out any more realizations than strictly necessary. For a clear illustration of this point, we again refer to Jaynes, who explicitly calculates this multiplicity for different assignments of probabilities to the die [17].

With this brief introduction, we hope that we have conveyed the general applicability of the principle of maximum entropy. In particular, in combination with Bayesian inference, it is a powerful tool for consistent reasoning in the light of new data. For the remainder of the paper, we focus on a particular problem in computational biology which has recently been the subject of substantial activity, in the pursuit of a practically workable maximum entropy solution to replace (or validate) the currently used approaches.

## Macromolecular Structure Determination

Molecular simulations typically utilize either molecular dynamics (MD) or Monte Carlo (MC) methods to sample conformations according to an energy function, 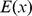. Here, 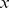 represents the structure of a molecule and possibly also solvent molecules and other co-factors, and 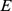 represents a mathematical function that relates the structure to the “energy” of the system. In the context of physics-based simulations, 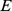 mimics the physical energy of the system; in such cases 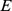 is also often referred to by its derivative, and thus called a molecular mechanics “force field” (herein termed 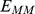). When MD or MC methods are used to sample protein conformations they typically give rise to an *ensemble* of conformations that are distributed according the celebrated Boltzmann distribution, 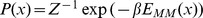, that relates the probability of observing a given conformation to the energy of that conformation. In this equation 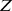 is a normalization constant and 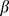 is the Boltzmann factor.

### Traditional structure determination methods

Despite recent substantial developments in the accuracy of molecular energy functions [18]–[20] it is still not possible routinely and consistently to use molecular simulations to predict or refine the structure of proteins [21], [22]. Protein structures are therefore typically determined through hybrid methods that combine experiments and simulations. The most common technique is to combine a physical force field, 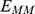 with an experimentally derived “biasing potential,” 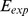: 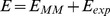. The function 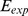 acts to bias simulations to provide structures that are compatible with experiments and typically takes the form of a harmonic potential that penalizes protein structures that are not in agreement with experiments: 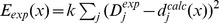. Here, 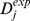 is the set of experimental measurements (e.g., NMR-derived NOE intensities or structure factors from X-ray scattering); 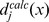 are the corresponding calculated quantities calculated from the structure, 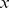, and 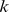 is a force constant that provides a scale for the energetic penalty for deviations between experimental and calculated values. In this way, only conformations where the back-calculated data are close to the experimental values will have a low overall value of 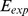. When combined with a force field, this produces structures that simultaneously agree with the experimental data and have structural features that conform to our current understanding of the physical principles that govern protein stability, encoded in 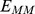 (e.g., well-packed hydrophobic cores and regular secondary structural elements stabilized by hydrogen bonds). When implemented in this fashion, it is important to note that the simulations are not forced to agree perfectly with the experimental data. Instead, the level of agreement is now governed by the weight of the biasing energy term. Consequently, the experimental data is typically referred to as *restraints*, rather than the term *constraints* used when complete agreement is the goal. One of the challenges associated with these hybrid energies is choosing such weights and other parameters for the biasing potential. Often these parameters are tuned manually. An alternative, Bayesian approach, called inferential structure determination, however, provides an elegant solution to this problem, by treating such unknown quantities as “nuisance parameters” and integrating them out [23], [24].

A direct consequence of the hybrid energy approach described above is that all of the sampled structures are *individually* in agreement with the experimental data. Although this superficially sounds reasonable—indeed the idea of the biasing potential is to bring the conformations to be in agreement with experiments—it brings with it some additional consequences. The basic problem arises because the experimental data, 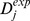, are typically averaged over a very large number of molecules as well as averaged over timescales that are long compared to those typical of macromolecular fluctuations. Thus, there is no reason to expect that individual conformations should be in exact agreement with the data as long as the entire ensemble of conformations is (e.g., even if a biased die produces an average of four, one would not expect that each throw produced this result). Thus, in the approach outlined above for structure determination, one is effectively making additional assumptions about both the structure and the conformational variability of a protein that are neither directly derived from the experimental data nor from the physical force field.

### Simulating replicas

One intuitive strategy to overcome this problem is to simultaneously simulate several replicas of the system and apply restraints on the *average* of the back-calculated experimental values, rather than on the individual structures [25]. This strategy has a long history and is often known as “ensemble averaged refinement” in the context of NMR structure determination [26], [27] or “multiconformer refinement” in X-ray structure determination [28]–[30]. This approach has, for instance, been used to study the structural dynamics of folded proteins [31]–[33], unfolded proteins [34], membrane proteins [35], and intrinsically disordered proteins [36].

In such ensemble simulations, the 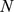 replicas do not physically interact, but are coupled via the experimental data. The total system of 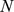 conformations is governed by the energy function:
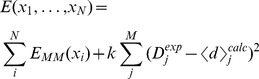
(1)


The first term is simply the sum of the force field energies of the replicas. The second term acts to enforce that the simulation is in agreement with the experiments, but penalizing the entire ensemble only when the *ensemble averaged* quantities, 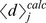 deviate from experiment. For linearly averaged quantities, 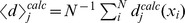. In this way, the calculated quantities in individual conformation (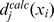) may differ from experiment as long as their ensemble average, 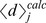, matches the experiment within a scale that is implicitly determined by the force constant, 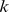.

Obviously, this method introduces a new parameter to the problem, namely the number of parallel replicas, and it is not immediately clear what this parameter should be set to. One approach to explore this problem is first to use synthetic data that have themselves been generated from simulations and compare the restrained ensemble with the ensemble used to generate the data [37], [38]. With real-world experimental data, a suitable value of 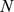 can be determined by cross-validating with independent data not used in the structure determination [39]. Since 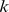 and 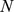 both affect the level of agreement between experiment and simulation, their optimal values are interdependent.

One critique of the method relates to the ratio of the number of free parameters (atomic coordinates) to the number of experimental data points. In “normal” (non-ensemble) structure determination, there are typically fewer experimental data than atomic positions to be determined; the problem is underdetermined and additional (prior) information, e.g., from a force field, is needed to determine structures. In ensemble refinement, the same number of data points are available, but now these are used to determine 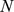 times as many atomic positions, leaving the underdetermination even worse. Although it can be argued that ensemble simulations provide a more natural way to match ensemble-averaged experimental data with simulations, the lack of a clear theoretical underpinning is problematic and it has been argued that the method can lead to an increased risk of drawing erroneous conclusions [40].

### Maximum entropy approach

Given the possibility of both overfitting experimental data and underrestraining by unfavourable data-to-parameter ratios, it would be preferable to have a theoretically well-founded method for combining experiment and simulation. Incorporating experimental data into a simulation is essentially a matter of updating a probability distribution (the original Boltzmann distribution defined by the force field) in the light of new data. As described above, these data are typically both time and ensemble averages of an underlying quantity, and it is therefore an obvious choice to use the principle of maximum entropy to infer a suitable model. Among all possible models compatible with the new data, this will be the one that is the least biased. Or alternatively phrased, this will be the model that is as close as possible to the original distribution, while taking the new data into account. As an important special case, we stress that if the force field is *already* compatible with the observed data, no modifications to the distribution are made. While this sounds like a trivial principle, it is actually violated by many existing methods.

Conceptually, the maximum entropy procedure is simple, and we proceed exactly as we did for the die example. The probability distribution takes the form
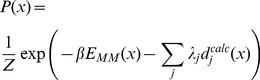
(2)where 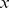 is the structural state, 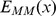 is the force field energy of this state, the 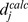 represent an experimental observable back-calculated from the structure, and 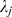 are the corresponding Lagrange multipliers, whose values should be determined to enforce agreement between experiment and simulations. Technical issues, however, seem to have hindered a practically useful implementation of the method. Although maximum entropy approaches have been explored in certain aspects of X-ray scattering data [41], [42] and NMR [43], [44], applications in the context of molecular simulation have been surprisingly few. One of the main practical issues is that one needs to numerically determine the optimal values for the Lagrange multiplier corresponding to each constraint. Since experimental data will easily provide hundreds of these constraints, this optimization is a formidable task.

In the last year, three papers have brought a practical application of the maximum entropy principle for this problem considerably closer [9]–[11]. Pitera and Chodera made the intriguing observation of a potential link between the maximum entropy solution and the solution obtained by the replica-averaged ensemble technique described above, suggesting that as the number of replicas in a simulation grows, the ensemble-restrained solution would gradually approach that obtained by the principle of maximum entropy [9].

The relationship between replica-based simulations and the maximum entropy formalism was clarified and mathematically proven in papers by Roux and Weare [10] and Cavalli et al. [11], both of which demonstrated that a replica-based approach is equivalent to the maximum entropy solution. In addition to establishing this link, their result provides a solution to one of the primary problems associated with the maximum entropy problem in molecular simulation: the challenge of estimating the Lagrange multipliers numerically. In particular, they showed that the maximum entropy solution appears as a limit of the replica method when the harmonic potential enforcing the replica-averaged restraint becomes infinitely narrow. More precisely, *the distribution of each replica in a replica-averaged ensemble simulation (* *Eq. 1* *) will approach the maximum entropy distribution if both* 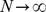 *and* 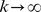. Using Dirac's 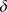-function over the averaged restraint violations, this can be written as
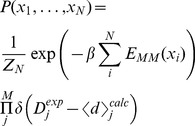
(3)As above, 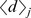 denotes the average ensemble average over the 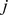
'th restraint, and 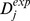 is the experimentally observed data.

**Figure 2:**
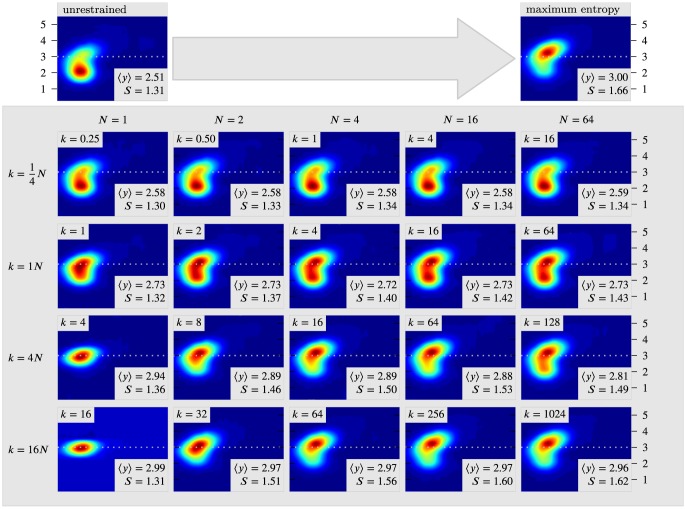
The effect of different methods for incorporating experimental data on a simple example consisting of a mixture of two bivariate normal distributions. In this example, we only have experimental data regarding the y-dimension of the distribution (target value indicated by dotted line). The top row contains the unperturbed and maximum entropy distributions. The matrix shows various combinations of force constant (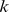) and number of replicas (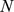) when enforcing the restraint through a harmonic potential. In these calculations, 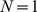 corresponds to the standard method for structure calculation, and 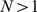 corresponds to ensemble refinement. In each plot we also show the mean in the y-direction (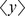), and the entropy of the distribution (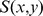).

This result has immediate practical applications. Rather than determining Lagrange multipliers for all experimental observations, it is sufficient to conduct an ensemble-averaged simulation with 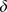-function constraints with a large number of replicas. In practice, 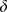-functions are difficult to work with and are often replaced with a steep potential, for instance a harmonic term. Figure 2 illustrates the procedure on a simple 2D bivariate Gaussian mixture model with two components, with a single restraint in one of the dimensions (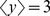). The top-left plot is the unperturbed potential, while the top-right plot shows the maximum entropy solution with a numerically optimized Lagrange multiplier. The plots in the matrix show the behavior of different combinations of the force constant of the harmonic potential and the number of replicas used in the simulation. Note how two opposite forces are at play: an increase in the force constant 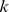 will pull the distribution toward the restrained value, while an increase in 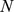 will increase the variance, pulling it back to the original distribution. For sufficiently large values of 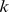 the harmonic term mimics a 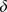-function and when 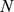 is increased for such values of 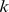 the distribution converges towards the maximum entropy solution *without explicitly determining any Lagrange multipliers*.

### Remaining challenges

The replica-averaged approach described in the previous section is a remarkably elegant, easily implementable technique that provides the least-biased distribution consistent with any observed expectation values over the data. From our perspective, it represents a significant step forward in our understanding of how experimental data should be used in molecular simulations. There are, however, still some remaining issues, which must be resolved before we can claim a full understanding of the problem and a practically useful implementation. We will highlight the most important ones here.

#### Ensuring convergence

There are some challenges involved in determining optimal values for the number of replicas 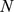 and the force constant 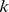. The question of how quickly 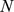 and 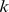 should grow with respect to each other was investigated in some detail for the 1D harmonic system by Roux and Weare, who stressed the importance of letting 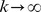 more rapidly then 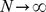 [10] but also noted that the exact details could differ for more complex models. Ideally, 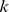 should be chosen as large as possible, but as illustrated by Figure 2, this will impose a requirement for a larger number of replicas as well. This observation suggests an iterative approach of alternating increases of 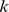 and 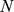. Higher values of 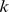 will draw the mean value closer to the restraint, while increasing values of 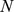 will increase the variance.

Convergence can be assessed by simultaneously probing the violation of the expectation values relative to the restraints and the entropy of the distribution (or the entropy corresponding to the restrained subspace). It is, however, very difficult to get converged entropy estimates for the high dimensional conformational space of a molecular simulation [45]. It remains to be seen whether this poses a significant problem for the application of this method in practice.

#### Estimating Lagrange multipliers

Despite the convenience of the replica-averaged method, it remains unclear whether this method is always preferable to an approach that estimates the Lagrange multipliers explicitly. Although there can be hundreds of parameters to estimate, there are mitigating circumstances, such as convexity in the case of independent restraints, which might make the search problem less complex. Roux and Weare point out that even when successfully finding all Lagrange multipliers, one still has to run an entire simulation. Similar problems seem to assert themselves for the replica-case, where production runs can only be conducted once convergence in entropy has been ensured.

One potential compromise could be the 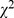 approach described below, which assumes that different restraints share the same Lagrange multiplier, and therefore requires fewer parameters to be estimated. Whether this approximation in practice proves more efficient than finding local-optima in the full restraint Lagrange problem remains to be seen. One direction that is worth pursuing further in this respect is to develop a replica analogy to the 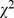 approach, alleviating the need for the numerical determination of the Lagrange multiplier.

#### Dealing with uncertainties

The previous sections assumed that the experimentally observed values were obtained with perfect accuracy. In any real-world scenario there will, however, be some level of noise or uncertainty associated with such experimental data. As an example, consider the case of the die in Box 1: the experiment from which the averages are observed will always consist of a finite number of tosses, and the average 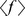 is therefore only determined within some uncertainty. How should this uncertainty be taken into account? Note that there is an important difference between adding a constraint on the second moment (the variance) and incorporating knowledge about the error of the first moment (error of the mean). The former can easily be dealt with using the maximum entropy principle, while the latter is more problematic.

One potential solution to the problem is to replace a single, exact constraint with two constraints that act as a lower and upper bound, respectively [10], [46]. This solution, however, assumes that the experimental noise can be interpreted as “hard” limits and does not represent the fact that the experimental measurement is just an estimate of the underlying true value.

As an alternative solution to this problem, Cavalli et al. propose a combination of the maximum entropy principle and Bayesian inference with a prior distribution that reflects the uncertainty of the measured quantity. We briefly sketch the idea behind the resulting derivation here, referring to the measured data points as 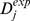 and the (unknown) actual values as 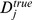. For compactness, boldface is used to denote a sets of replicas or restraints (e.g., 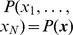). Assuming independence between the restraints, we have:
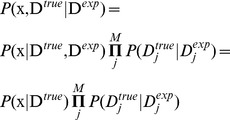
(4)Assuming flat priors, we have 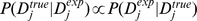, and assuming independent Gaussian distributions on the latter,
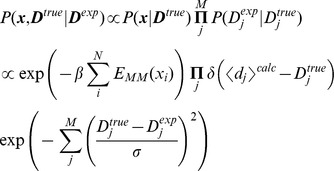
(5)where 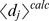 again is the calculated ensemble averaged quantity of data 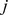. Note how this is simply the product of a noise-free maximum entropy expression on the exact but unknown quantity 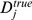 and a noise term that models the uncertainty of our observable 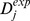. 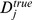 are “nuisance parameters” that can now be integrated out:
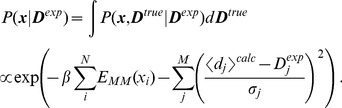
(6)This expression now only includes 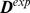, the experimentally determined quantity that is an estimate of the true, underlying value. The equation above, derived by Cavalli et al. is quite striking, in the sense that it corresponds exactly to the form used in classic ensemble simulations (Eq. 1), except that the force constant that can normally be tuned freely is now determined uniquely by the uncertainty in the observed experimental values.

While this expression is highly appealing, it also presents a potential complication: because the force constant is now fixed (by the experimental uncertainty), the replicas will decouple in the limit of 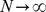, and the influence of the data will decrease as the ensemble approaches the unperturbed distribution provided by the force field. In the context of the example in Figure 2, it is clear that if the force constant is too low, such as in the first row, increasing the number of replicas does not lead to a distribution that mimics the maximum entropy solution. While it is clear that in the presence of experimental noise one would not expect to recover the standard maximum entropy result (which is valid only for exactly known quantities), one would expect that the experimental uncertainty, 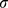, should set the scale for how large deviations can be tolerated between the final ensemble and the experimental value. The effect can also be understood in the detailed analysis by Roux and Weare of a 1D harmonic potential with a harmonic restraint. In particular, their calculations show that when the number of replicas is increased for a fixed force constant, the mean of the restrained ensemble converges to that of the prior reference distribution while the variance increases to its correct value. In the standard maximum entropy setting, the problem of the mean reverting to the reference value can be alleviated by simply increasing the force constant. When the force constant is determined from the experimental noise, this is no longer possible, suggesting that rather than the 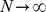 limit, an intermediate value of 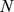 might be more appropriate in order to provide a balance between matching the mean and the variance. Although this leaves open exactly which procedure is most appropriate to determine the optimal value of 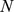, it does provide insight into the problems associated with choosing a value that is either too low or too high.

The problem of maximum entropy in the context of noisy data has been addressed numerous times in other fields, leading to various forms of generalized maximum entropy procedures [46], [47] and regularization approaches [48], [49]. Unfortunately, as of yet there seems to be no universally accepted solution to this problem. One approach that we foresee could be potentially useful in molecular simulation was proposed by Gull and Daniell in the context of image reconstruction [50]. The idea is to replace the many individual constraints with a single constraint on the 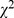 statistic over all data, only matching them up to their experimental uncertainty:
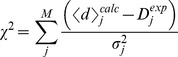
(7)The expectation of this statistic is the number of data points 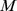. Maximizing the entropy with respect to this single constraint we obtain:
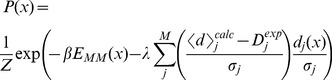
(8)This approach only requires a single Lagrange multiplier to be determined (by matching the calculated 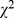 with its expectation value) and, thus, scales considerably better with the number of observed data points. The resulting expression, however, relies on the averages 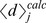, which are not know a priori. A possible strategy would be to estimate 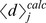 and 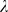 iteratively, by repeatedly estimating 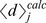 from a simulation, adjusting 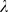 to match the calculated and expected value for 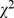, and then rerunning the simulation (or reweighting the statistics from the previous one [8]). It might also be possible to use ensemble simulations to provide an initial ensemble to help determine 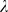. To our knowledge, this method has not yet been applied to molecular simulation, and the practical applicability of the approach therefore remains to be established.

Finally, we note that an alternative approach has very recently been suggested to derive structural ensembles from noisy, ensemble-averaged experimental data [51]. This method is an extension of the Bayesian inferential structure determination method that includes ensemble averaging via a hierarchical model, and it attempts to find an ensemble that is least biased compared to prior knowledge (e.g., a force field) and that simultaneously is compatible with the experimental data.

## Discussion

The three new papers highlighted in this Perspectives article have provided substantial new insights to the field of molecular simulation under experimental restraints. Of particular interest is the result that the current common practice of replica-averaged simulations is tightly linked to the solution prescribed by the maximum entropy formalism. This link provides an attractive way forward for the field. Replica-averaged simulations have a substantial track record and have in many cases been shown to improve the quality of structural ensembles—e.g., measured through cross-validation with unrelated experimental data—and to provide new biological insights. The relationship with the maximum entropy solution suggests that the restrained ensemble can be regarded as the proper thermodynamic ensemble that represents a system when both the energy and some additional experimental data are known. As such, the system-specific force field correction introduced by the restraints, when applied appropriately, may be viewed as a natural extension of the Boltzmann ensemble when one is provided with additional information beyond the energy.

In addition to the theoretical developments highlighted in this article, an important area for future studies is how best to implement them in practice. In the case of data without noise it is, for example, not clear how many replicas are needed in practice to recover an ensemble that is close to the maximum entropy solution. Another question is related to the steepness of the potential used to implement the restraint: how narrow should the potential be to mimic the appropriate 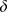-function that will ensure the maximum entropy correspondence? Previous work has either found optimal values of the number of replicas by cross-validation, or simply chosen a sufficiently large 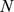 to obtain convergence. As both illustrated by theoretical results [10], [11] and our own simulations (Figure 2), it is, however, necessary to choose the force constant sufficiently large to converge to the maximum entropy solution. The results also show that that one can reach apparent convergence at lower values of the force constant, but that the resulting distribution in this case will not be the maximum entropy solution. For these and related problems, we also need better methods to check for convergence, both to study the effect of varying these restraint-parameters and to monitor and ensure sufficient sampling of the ensembles.

Another topic that remains incompletely understood is how best to deal with uncertainties in the observed data. Cavalli et al. provide a possible path in this direction, and in this paper, we have sketched out a few potential alternatives. From a theoretical viewpoint, it seems desirable to combine Bayesian inference, which provides a robust toolbox for dealing with noisy data, with the maximum entropy principle for deriving probability distributions in underdetermined systems. There already exists a large literature on these topics in other disciplines, but further studies and applications on real systems are necessary to shed further light on which methods are most useful in biological simulations.

As we have here hinted, the problem of uncertainties in the data appears to be related to the problem of determining the relative weight between force field and restraint-potential. A relevant question in this context is whether such a weight can be meaningfully defined and assigned without considering the inherent accuracy of the force field itself. Intuitively, if the force field in question is a preliminary implementation, it should be weighed lower than if it has been carefully parameterized against a large amount of data. This degree of trust is currently not encoded in the force fields commonly employed in simulations. In principle, this information could be specified by providing distributions (or at least variances) for all estimated parameters in the force field, in the spirit of Bayesian inference, allowing the inference machinery to deduce or integrate out the relevant weights. In this way, a force field would no longer be characterized by a single set of parameters, but instead as a “distribution of force fields.” There are significant challenges associated with estimating and sampling from such models, but recent work provides hope for the eventual feasibility of such an approach. First, advances in techniques for force field optimization [8], [52] allow for a Bayesian approach to integrate experimental data and, e.g., quantum-level data, bringing us closer to the ability to probe the uncertainties associated with individual parameters. Second, on the sampling front, inferential structure determination has demonstrated how (small numbers of) parameters can be successfully integrated out during a simulation [23], [24], [53]. Thus, we envisage that in future applications it might be possible to integrate out not only experimental noise and “nuisance parameters,” but potentially also the uncertainty associated with the parameterization of a force field. We note that distributed computing platforms may be particularly well suited to sample from such models as one might need to perform multiple, independent simulations that differ only slightly in the force field used.

We also point out that ensemble simulations inherently have more unfavourable data-to-parameter ratios than standard methods for structure determination. As such, they may in particular benefit from improved force fields, and we expect that as force fields continue to improve it should become possible to study more complex systems with less experimental information. Importantly, a consistent theoretical framework should allow us to transition smoothly between traditional, mostly data-driven methods for structure determination and molecular simulations in the absence of any experimental data.

Finally, we note that although the developments described here have focused on restraining molecular simulations with experimental data, maximum entropy methods have a broad range of applications both in biology and beyond. We envisage that new theoretical developments, such as the link between ensemble simulations and maximum entropy solutions, can be directly applicable in other fields. Similarly, new methods for deriving modified models in the context of noisy data should have broad applicability. For example, the recent advances in predicting structural contacts from a maximum entropy–based analysis of the covariation of sites in a multiple sequence alignment [6] should benefit greatly from improved techniques for handling the uncertainty associated with limited sequence numbers.
